# Extraction Optimization of Flavonoids from *Hypericum formosanum* and Matrix Metalloproteinase-1 Inhibitory Activity

**DOI:** 10.3390/molecules22122172

**Published:** 2017-12-11

**Authors:** Ho-Shin Huang, Ean-Tun Liaw

**Affiliations:** 1 Department of Food Science, National Pingtung University of Science & Technology, Pingtung 91201, Taiwan; adinol.huang@gmail.com; 2 Testing Center of Cosmetic, Tridl Inc., Tainan 70955, Taiwan

**Keywords:** response surface methodology, *Hypericum formosanum*, matrix metalloproteinase-1, flavonoids

## Abstract

*Hypericum formosanum* is a valuable herb in Taiwan. In this study, response surface methodology was employed to optimize the ultrasound-assisted extraction of flavonoids from *Hypericum formosanum*. A central composite design with three variables (ethanol concentration, extraction time, and extraction temperature) was applied. Experimental results were fitted to the second order polynomial model and one-way analysis of variance was used to determine the goodness of fit of the model and the optimal conditions for responses. The optimal conditions for the maximum extraction yield of total flavonoid content (101.1 mg/g) using ultrasound-assisted extraction were ethanol concentration, 73.5%; extraction time, 38.3 min; and extraction temperature, 62.5 °C. The predicted result was consistent with the experimental result obtained under optimal extraction conditions. Hyperoside, astilbin, quercitrin, and quercetin from *Hypericum formosanum* extract (HFE) were identified by Ultra performance liquid chromatography-diode array detector-mass (UPLC-DAD-MS). HFE significantly reduced matrix metalloproteinase-1 protein expression in human skin keratinocyte cells, induced by advanced glycation end products.

## 1. Introduction

In various parts of the world, plants of genus *Hypericum* have been used in traditional medicines, as an antiseptic, and as an antispasmodic, as well as in the treatment of external wounds and gastric ulcers [1]. Plants of the genus *Hypericum* spp. are a rich source of flavonoids and exhibit a broad spectrum of activities. The flavonoids and their derivatives, isolated from different species of *Hypericum*, exhibit potent anti-tumor, anti-fungal, anti-microbial [2,3,4], anti-ulcer, anti-depressant, antioxidant, and anti-inflammatory activities [5,6,7]. *Hypericum formosanum* is native to Taiwan and is a valuable folk herb in Taiwan. However, to the best of our knowledge, there are no reports in literature on the extraction of flavonoids from *Hypericum formosanum*.

In recent years, many studies have focused on extraction optimization of flavonoids and their health and medicinal applications [8,9,10]. Ultrasound assisted extraction (UAE) is established as a useful technique for the extraction of polyphenol and flavonoid compounds from plant sources, and has been found to be more effective than other extraction methods [11,12,13]. Moreover, recent studies have demonstrated the effects of several parameters on the extraction of flavonoid compounds from some herbs, using response surface methodology (RSM) [14,15,16]. RSM is widely employed as an effective modeling tool to construct functional relationships between a response variable and design variables. Several studies on conditions optimized for the extraction of phenolic compounds from different sources using RSM have been published [17,18,19,20]. Central composite design (CCD) is the most popular form of RSM and has been widely used for analysis to optimize various parameters in extraction conditions [21]. The efficiency of extraction depends on several parameters, such as temperature, time, and solvent polarity, and their effects can be either independent or interactive [22,23]. In this study, UAE was used to extract flavonoids from the whole plant *Hypericum formosanum*, and the effects of ethanol percentage, extraction time, and extraction temperature on the total flavonoid content (TFC) were studied. Extraction conditions were then optimized using response surface methodology.

The process of skin aging can be divided into intrinsic and extrinsic categories. Intrinsic aging is caused by the aging process and is highly correlated with genetic factors. However, extrinsic aging is mainly due to exposure to ultraviolet (UV) light and is characterized by severe wrinkling and collagen degradation [24,25]. Glycation resulting from UV light exposure, genetic factors, and environmental pollution promotes skin aging [26,27]. Glycation causes advanced glycation end products (AGEs) to accumulate in the skin during skin aging, especially in long-lived proteins such as dermal elastin and collagen [28,29]. Matrix metalloproteinases (MMPs) are zinc-dependent endopeptidases that are important in the remodeling of extracellular matrix (ECM) structure in skin. MMP-1 is a collagenase, and plays an essential role in the physiological mechanism of skin aging [30]. AGEs could increase the expression of MMP-1 in human keratinocyte cells. It has been reported that flavonoids have inhibitory effects on glycation processes and cause decreased MMP-1 expression [31]. Flavonoids are efficient antioxidants that possess anti-cancer and anti-aging activities [32]. In our study, we found quercitrin was the major compound in the extract from *Hypericum formosanum*. Quercitrin is a bioflavonoid with antioxidant properties and is better absorbed than other forms of quercetin [33]. Additionally, quercitrin showed excellent inhibitory effects on AGE formation and good anti-glycation activity [34,35]. In this study, the inhibitory activities of the *Hypericum formosanum* extract and quercitrin on MMP-1 were also evaluated.

## 2. Results and Discussion

### 2.1. Effects of Time, Ethanol Concentration, and Temperature on the Extraction of Flavonoids

**Figure 1:**
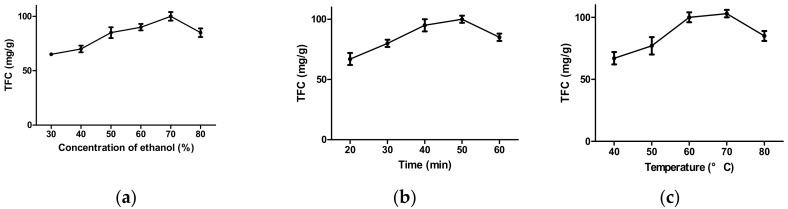
The effects of (**a**) concentration of ethanol; (**b**) extraction time; and (**c**) extraction temperature on the total flavonoid content (TFC) of the extract (*N* = 3).

To examine the effects of ethanol concentration on the extraction of flavonoids, a range of 30–75% solvent concentration was tested in this study. Our results revealed that ethanol concentrations of about 70% resulted in the highest flavonoid value (Figure 1a) and that flavonoid extraction yield was greatly influenced by the concentration of ethanol. The reasons for this might be related to the polarity of ethanol and the solubility of flavonoids in *Hypericum formosanum.* Figure 1b shows that flavonoid values increased as extraction time increased from 40 to 50 min. After extraction time exceeded 50 min, the extraction rate dropped. Longer extraction times may enhance extraction efficiency, but may also increase the oxidation of phenolic compounds [21].

Extraction temperature was another important factor for the extraction of flavonoids from herbs. This study showed, as shown in Figure 1c, the TFC of the *Hypericum formosanum* extract (HFE) increased with an increase in temperature up to 70 °C, with a maximum TFC at 60–70 °C. These results are explained by the fact that the release of bioactive compounds from plant cells can be increased by an increase in extraction temperature, but temperatures higher than the optimized level may cause the decomposition of several thermo-sensitive compounds within the extract [36].

### 2.2. Fitting the Model and Response Surface Analysis

Based on the procedures for central composite design experiments, the values of the independent variables were chosen based on the values obtained from the single-factor experiments. Ethanol concentration (*A*, %, *v*/*v*), extraction time (*B*, min), and extraction temperature (*C*, °C), which have a significant effect on the extraction rate of total flavonoids, were selected as design variables for the RSM test. The RSM test was designed using the extraction rate of TFC (mg/g) as the response variable (Table 1). Results showed that the TFC values ranged from 65.5 to 101.5 mg/g dry weight (DW). A quadratic polynomial model using multiple regression analysis could describe the results of the CCD. One-way analysis of variance (ANOVA) for the fitted equation is illustrated in Table 2. The *F*-test was conducted to check whether the regression equation was statistically significant. The *F*-value was high (28.33) and the *p*-value was low (<0.0001), which implied the model obtained was statistically significant. In addition, the determination coefficient value (*R*^2^) was 0.9587, which implies strong correlation between the predicted results and the actual results. In addition, the lack of fit was not significant (*F* = 2.89; *p* = 0.1342 > 0.05), indicating that variation is predicted by the model [23]. These data revealed that the model was appropriate for forecasting TFC values of HFE within the tested ranges. In this model, the linear parameter X_1_ was significant at the level of *p* < 0.0001, and the interaction parameter X_1_X_2_ and quadratic terms X_2_^2^ and X_3_^2^ were significant (*p* < 0.05) for the extraction yield of flavonoids. The linear parameters X_2_ and X_3_, the interaction parameters X_1_X_3_ and X_2_X_3_, and the quadratic term X_1_^2^, were not significant (*p* > 0.05). After discarding the insignificant parameters, the regression model was modified as below:

*Y* = 93.28 + 10.17*A* + 1.78*B* + 3.25*C* + 0.98*AB* + 0.33*AC* − 1.02*BC* − 1.26*A*^2^ − 4.41*B* − 9.36*C*^2^

**Figure 2:**
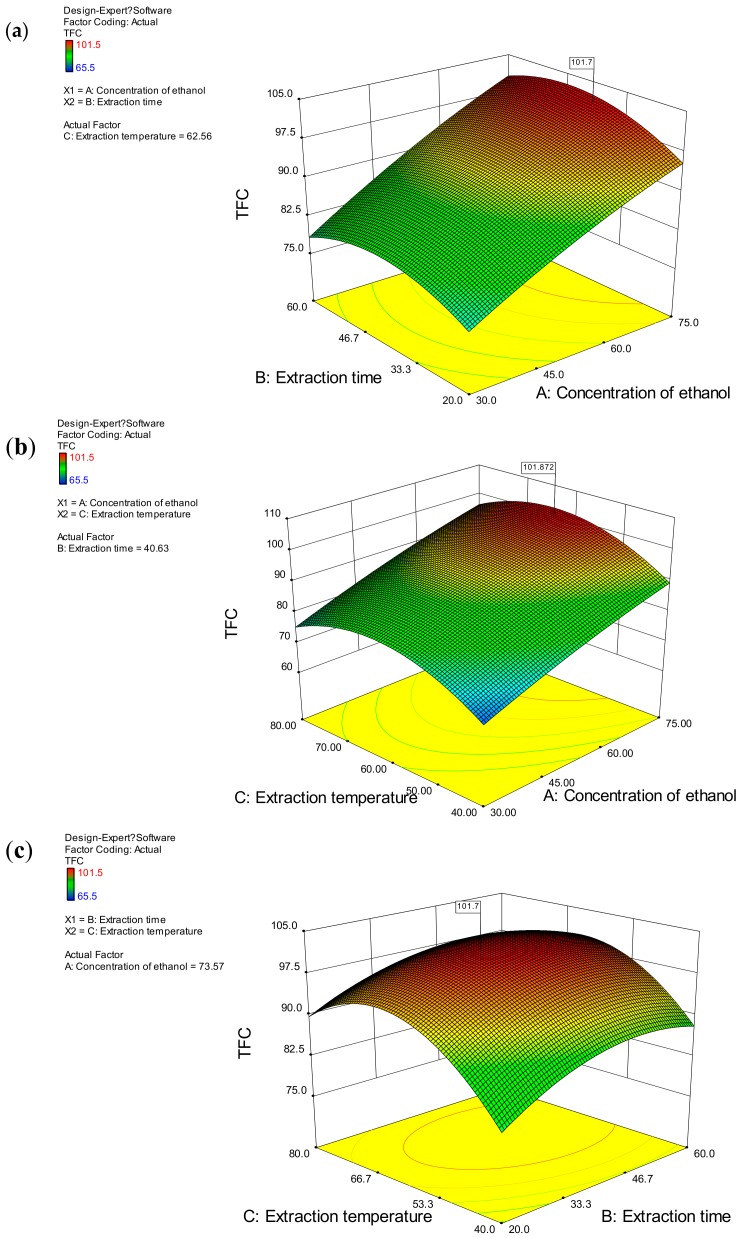
Response surface plots (3D) showing the effects of different extraction parameters including (**a**) concentration of ethanol; (**b**) extraction time and (**c**) extraction temperature on the response.

**Figure 3:**
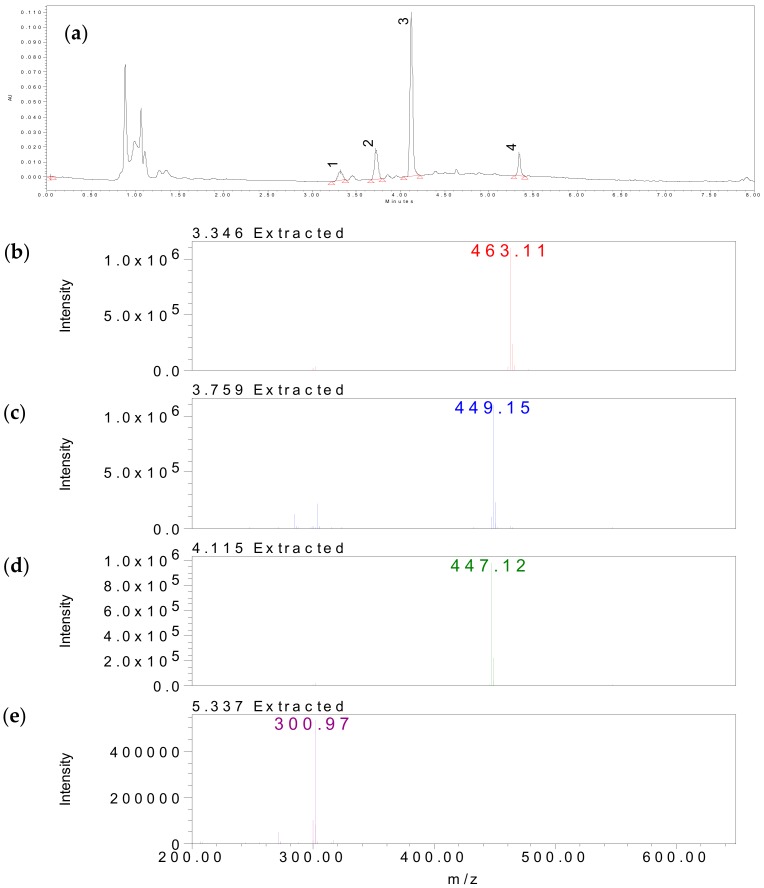
UPLC-DAD chromatograms obtained at 270 nm from *Hypericum formosanum* extract (HFE); (**a**) 1 = Hyperoside, 2 = Astilbin, 3 = Quercitrin, 4 = Quercetin; (**b**) MS of Hyperoside; (**c**) MS of Astilbin; (**d**) MS of Quercitrin; (**e**) MS of Quercetin.

The graph of RSM was a 3D response surface plot consisting of response values of the experimental variables (Figure 2), which reflects the interactions between the variables (ethanol concentration, extraction time, and extraction temperature). The combined effect of ethanol concentration and extraction time was not significant; as can be seen in Figure 2a the effect of extraction time on the extraction rate of flavonoids was not very obvious at a given ethanol concentration as the surface is relatively flat. Figure 2b shows the 3D response surfaces for the combined effect of ethanol concentration and extraction temperature on TFC, and it reveals that at low ethanol concentration and high extraction temperature values, the TFC was minimal. When extraction temperature was at a certain value, the TFC increased with an increase in ethanol concentration. However, it was not significant that the increase in the extraction temperature affected the TFC at a certain ethanol concentration. As shown in Figure 3c, extraction time and extraction temperature demonstrated a quadratic effect on the response. Based on the results of the response surface plots and ANOVA, it is obvious that ethanol concentration was the main parameter influencing the TFC value.

### 2.3. Optimization and Verification

By using Design Expert 8.0 software (Stat-Ease, Minneapolis, MN, USA), the optimum conditions were obtained. The optimal conditions for the maximum extraction yield of total flavonoid content (101.1 mg/g) using ultrasound-assisted extraction were ethanol concentration, 73.5%; extraction time, 38.3 min; and extraction temperature time, 62.5 °C. The results showed that the experimental values (101.7 mg/g) were not only consistent with the predictive values (Table 3), but were also better than any single factor experiments. Therefore, the extraction conditions obtained by RSM were not only accurate and reliable, but also of practical value reflecting the expected optimization. This study shows that the extraction of flavonoids from *Hypericum formosanum* can be improved by optimizing extraction parameters.

### 2.4. Identification of Bioactive Compounds by HPLC-MS Analysis

Medicinal plant extracts and isolated secondary metabolites have traditionally been used to treat a wide variety of diseases with acute and chronic inflammation. Fruits and vegetables are important anti-inflammatory compounds due to their potent anti-oxidative activity and this is especially true for flavonoids in herbs [37]. In addition, flavonoids are often involved in the pharmacological activity of important medicinal plants. In previous studies, plants of the *Hypericum* genus have been shown to contain many bioactive compounds, including phloroglucinols, naphthodianthrones, flavonoids (hyperoside, rutin, quercetin, quercitrin, isoquercitrin, miquelianin, astilbin), and phenolic acids (chlorogenic acid) [5,6]. In this study, the high performance liquid chromatography (HPLC) chromatographic profile of *Hypericum formosanum* extract was shown in Figure 3a–c. The major compounds of *Hypericum formosanum,* including hyperoside (1), astilbin (2), quercitrin (3), and quercetin (4), were identified by Ultra performance liquid chromatography-diode array detector-mass (UPLC-DAD-MS) analysis and comparing their mass data with standards.

### 2.5. Matrix Metalloproteinase-1 Inhibitory Activity 

**Figure 4:**
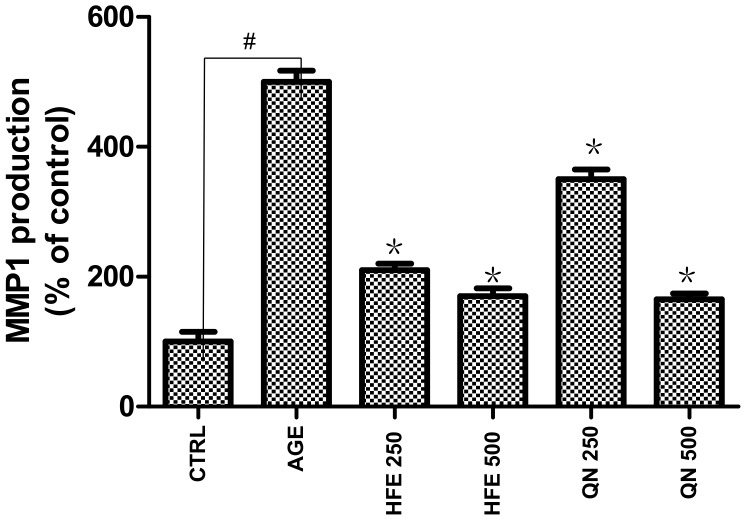
Effects of HFE and QN (quercitrin) on Matrix metalloproteinase 1 (MMP-1) expression in human skin keratinocyte (HaCaT) treated by advanced glycation end products (AGEs) for 24 h. Data are shown as the mean ± standard deviation (SD) of three independent experiments (Positive control: 24 h AGE-induced #: *p* < 0.05 compared to negative control; *: *p* < 0.05 compared to positive control by Student’s *t*-test).

Cell viabilities were measured by 3-(4,5-dimethylthiazol-2-yl)-2,5-diphenyltetrazolium bromide (MTT) assay. Treatment of Human skin keratinocyte cells (HaCaT cells) with HFE and quercitrin at various concentrations from 250 to 500 μg/mL for 24 h exhibited that cell viability was more than 90%. MMP-1, also called collagenase-1, is chiefly responsible for the degradation of type I collagen, a primary component in and structural support for the skin dermis [29]. It is believed that the flavonoids in herb extracts play an important role in MMP inhibition and other anti-proliferative effects [38]. Our study found AGEs raised MMP-1 expression in HaCaT cells (Figure 4). Data revealed that HFE and quercitrin treatment at 250–500 μg/mL markedly down-regulated MMP-1 in this cell line (*p* < 0.05). The level of MMP-1 protein decreased significantly by 58 ± 1.2% and 66 ± 2.3% compared to the control level at concentrations of 250 μg/mL and 500 μg/mL of HFE, respectively. As shown in Figure 4, quercitrin suppressed AGE-induced MMP-1 expression by 30 ± 1.7% and 67 ± 2.0% compared to the control level at the concentrations of 250 μg/mL and 500 μg/mL, respectively. Some studies have found that flavonoid compounds from herbs provided significant inhibitory effects on UVB-induced matrix MMP-1 expression in HaCaT cells [39]. Similarly, we found quercitrin shows significant activity, indicating that quercitrin plays an important role in MMP-1 inhibitory activity. In addition, the inhibitory activity of HFE, which contains multiple compounds, was better than quercitrin at 250 μg/mL. Some studies have suggested herbal extracts show stronger and more effective bioactivity effects than any of the single compounds due to the coordination of numerous natural compounds [40,41]. Results from our study also showed similar results; that the multiple compounds within herb extracts might increase MMP-1 inhibitory activity by a synergistic effect. However, further in vivo studies are necessary to elucidate its MMP inhibitory activity and synergistic effect mechanisms.

## 3. Materials and Methods

### 3.1. Plant Materials

The tissues of *Hypericum formosanum,* including the leaves, stem, and roots, were harvested. The plant material was identified by Dr. Chang, San-Xian, botanist of the Hualien District Agricultural Research and Extension Station Council of Agriculture. The plant materials were washed with water and air-dried at room temperature for 5 days. The sample was then oven-dried at 55 °C for 2 days and ground to a fine powder by an electronic blender.

### 3.2. Chemicals

Methanol, ethanol, acetonitrile, formic acid, aluminium chloride (AlCl_3_), sodium nitrite (NaNO_2_), sodium hydroxide (NaOH), and Advanced glycation end products (AGEs) were purchased from Merck Co. (Darmstadt, Germany). Dimethyl sulfoxide (DMSO), streptomycin, phosphate buffer saline, 3-(4,5-Dimethylthiazol-2-yl)-2,5-diphenyltetrazolium bromide (MTT), catechin, hyperoside, quercitrin, quercetin, and astilbin and were purchased from Sigma Co. (St. Louis, MO, USA). Fetal bovine serum (FBS) and Dulbecco’s Modified Eagle’s Medium (DMEM) were purchased from Gibco (Invitrogen, Carlsbad, CA, USA).

### 3.3. Plant Materials and Preparation of the Extracts

The powder of *Hypericum formosanum* tissue was extracted with ethanol using an ultra-sonicator. For ultrasound-assisted extraction, a sonication water bath (Delta, DC-400H, Taipei, Taiwan with a frequency fixed at 40 kHz was used. In experimental runs, 0.5 g of *Hypericum formosanum* powder was mixed with 50 mL of ethanol at various concentrations (according to the study design) in 100 mL flasks. After extraction, the extracts were instantly filtered through the filter paper under reduced pressure. Extracts were collected into glass flasks and stored at 4 °C until further analysis.

### 3.4. Total Flavonoid Content

Total flavonoid content (TFC) was determined based on Kim’s method [42] with some modifications. One mL of diluted extract solution (1 mg/mL) was mixed with 0.3 mL of 5% NaNO_2_ and 4 mL of distilled water. 0.3 mL of 10% AlCl_3_ was added to the mixture followed by adding 2 mL of 1 M NaOH. The solution was immediately diluted to 10 mL using distilled water. The absorbance of the solution was measured at 510 nm. Results of total flavonoid content were expressed as g of catechin equivalents (CE) on dry weight of dry extract (mg CE/g DW). All experiments were performed in triplicate.

### 3.5. Single-Factor Experiments

In order to investigate the influence of every factor on the TFC of the *Hypericum formosanum* extract, single-factor experiments were conducted as to determine the effects of the five different factors. Concentration of ethanol (30–75%), extraction time (20–60 min), and temperature (30–60 °C) were determined on the basis of the TFC yield.

### 3.6. Experimental Design

To evaluate the effect of each factor on the total flavonoid content of the *Hypericum formosanum* extract, a single-factor experimental design was first adopted for analyzing the influence of the three different variables. The effects of the concentration of ethanol, extraction temperature, and extraction time were investigated. Optimization of the UAE of TFC from the *Hypericum formosanum* was further carried out using RSM [22]. A three-factor and three-level CCD consisting of twenty experimental runs was employed at the center point (Table 4). The experimental data were fitted to the second-order polynomial equation given below:

*Y* = β_0_ + Σ βi Xi + Σβii Xi^2^ + Σ βij Xi Xj (1)

where *Y* is the predicted response (TFC), and β_0_, βi, βii, and βij are the regression coefficients for the intercept, linear, quadratic, and interaction terms, respectively. Xi and Xj are independent variables.

The design of experiments, analysis of the results, and prediction of the responses were performed using Design-Expert software 8.0 (Stat-Ease, Minneapolis, MN, USA). Comparisons of the means were performed using one-way analysis of variance. The extraction conditions were optimized for the maximum yield of TFC based on the regression analysis and the 3D surface plots of the independent variables. Finally, the predicted values were compared with the experimental values to determine the validity of the model.

### 3.7. UPLC–DAD-MS Analysis of the Extract

The HFE samples, dissolved in methanol, were separated on an RP-C18 column (XBridge 2.5 μm, C18, 100 × 2.1 mm) by UPLC equipped with a Waters Acquity system consisting of a column oven, sample manager, solvent manager, and DAD detector (Waters Corp., Milford, MA, USA). The flow rate was 0.4 mL/min. The solvent gradient for UPLC was a mixture of 0.1% formic acid/H_2_O (solvent A) and 0.1% formic acid/acetonitrile (solvent B): 85% A from 0 to 3 min, 45–65% A from 3 to 5 min, and 45–0% A from 5 to 8 min. The column temperature was fixed at 40 °C. A 270 nm wavelength was employed to quantify the flavonoids. Identification of the constituents was carried out with a Waters Acquity QDa Mass Detector with an Electro Spray Ionization (ESI) source, and Empower software (Waters, Milford, MA, USA) was used for data analysis. Negative ion mass spectra were recorded in the range *m*/*z* 200–650. Probe temperature was kept at 600 °C and Cone Voltage at 30 V.

### 3.8. Cell Culture

A human skin keratinocyte (HaCaT) cell line from American Type Culture Collection (ATCC, Rockville, MD, USA) was cultured in Dulbecco’s Modified Eagle’s medium (DMEM) supplemented with 10% fetal bovine serum and 100 U/mL streptomycin under 95% air/5% CO_2_ at 37 °C. Culture medium was changed every three days, cells were sub-cultured once a week, medium was changed to serum-deprived medium, and cells were washed with serum-free DMEM 24 h before experiments and replanted in 96-well plates. Phosphate buffered saline (PBS, pH 7.2) was used to adjust cell number to 10^5^/mL for various experiments and analyses.

### 3.9. Cell viability Assay

Cell viability was analyzed using a 3-(4,5-dimethylthiazol-2-yl)-2,5-diphenyltetrazolium bromide (MTT) assay. HaCaT cells were seeded in the wells of 96-well plates and incubated with HFE (250, 500, 1000 μg/mL) and quercitrin for 24 h. After incubation, MTT solution was treated for 4 h. The culture medium was removed and the formazan crystals that had formed were dissolved in DMSO and quantified at 570 nm using a microplate reader (MultiskanGO, Thermo Fisher Scientific Inc., Waltham, MA, USA).

### 3.10. AGEsTreatment and Real-Time PCR Analysis

HaCaT cells (10^5^ cells/mL) were treated with AGEs at 100 μg/mL for 24 h at 37 °C. After washing with serum-free DMEM, cells were exposed to *Hypericum formosanum* extract and quercitrin at 250 μg/mL and 500 μg/mL for 24 h. Control groups were HaCaT cells containing neither AGEs nor *Hypericum formosanum* extract. After 24 h mRNA was extracted from cells using Trizol Reagent (Invitrogen-Thermo Fisher Scientific, Waltham, MA, USA) according to the manufacturer. cDNA was obtained from 0.5–1 mg of total mRNA using Moloney Murine Leukemia Virus Reverse Transcriptase, MMLV-RT (Promega, Madison, WI, USA) according to the manufacturer. The mRNA relative expression was determined by real-time PCR using Fast Start Universal SYBR Green Master Mix (Roche, Basel, Switzerland) in a real-time PCR-System machine (Sure Tect, Thermo Fisher Scientific Inc., Waltham, MA, USA). The primer was MMP-1 (Forward: 5′-ATGCTGAAACCCTGAAGGTG-3′, Reverse: 5′-GAGCATCCCCTCCAATACCT-3′) and the internal control, GAPDH (Forward: 5′-CTCATGACCACAGTCCATGC-3′, Reverse: 5′-CACATTGG GGGTAGGAACAC-3′).

### 3.11. Statistical Analysis

Design Expert 8.0 was applied in the statistical analysis of the results in the RSM experiment. SPSS19.0 (IBM, Armonk, NY, USA) and Excel 2016 (Microsoft, Redmond, Washington, USA) were used for statistical analysis during the entire study.

## 4. Conclusions

In this study, a method by RSM optimization has been developed to extract flavonoids from *Hypericum formosanum*. The obtained regression model showed high correlation *(R*^2^ *= 0.9587*). Under the optimal condition of ethanol concentration 73.5%, extraction time 38.3 min, and temperature 62.5 °C, the predicted value for TFC was found to be 101.7 mg/g. The major compounds of *Hypericum formosanum,* including hyperoside, astilbin, quercitrin, and quercetin, were identified by UPLC-DAD-MS. The extract of *Hypericum formosanum* reduced AGE-induced MMP-1 protein expression. The major flavonoid compound in *Hypericum formosanum* could be correlated to its MMP-1 inhibitory activity. HFE may have medicinal value for applications in the cosmetics industry.

## Figures and Tables

**Table 1:** Factors and levels in the response surface central composite design arrangement and experimental results.

Run	X_1_	X_2_	X_3_	Response Value
	Concentration of Ethanol (%)	Extraction Time (min)	Extraction Temperature (°C)	TFC (mg/g)
1	75	20	80	90.1
2	52.5	40	60	89.9
3	30	20	40	65.5
4	30	40	60	80.2
5	30	60	80	70.5
6	75	20	40	81.9
7	52.5	40	60	94.7
8	75	40	60	101.5
9	52.5	40	60	94.5
10	52.5	40	80	90.2
11	52.5	40	40	75.3
12	30	20	80	70.2
13	52.5	60	60	90.2
14	30	60	40	67.7
15	75	60	80	92.1
16	52.5	20	60	85.2
17	52.5	40	60	95.2
18	75	60	40	90.2
19	52.5	40	60	94.9
20	52.5	40	60	95.1

**Table 2:** Analysis of variance for the quadratic polynomial model.

Source	Sum of Squares	df	Mean Square	*F*-Value	*p*-Value	Significant
Model	2084.65	9	231.63	28.33	<0.0001	Significant
X_1_	1034.29	1	1034.29	126.5	<0.0001	
X_2_	31.68	1	31.68	3.88	0.1234	
X_3_	105.62	1	105.62	12.92	0.0773	
X_1_X_2_	7.61	1	7.61	0.93	0.0049	
X_1_X_3_	0.84	1	0.84	0.1	0.3576	
X_2_X_3_	8.4	1	8.4	1.03	0.7545	
X_1_^2^	4.39	1	4.39	0.54	0.3345	
X_2_^2^	53.57	1	53.57	6.55	0.0284	
X_3_^2^	241.11	1	241.11	29.49	0.0003	
Residual	81.76	10	8.18			
Lack of Fit	60.77	5	12.15	2.89	0.1342	Not significant
Pure Error	20.99	5	4.20			
Core total	2166.41	19				
*R* ^2^	0.9587					

**Table 3:** The results of experimental verification.

Optimal Conditions			Total Flavonoid Content (mg/g)	
Concentration of Ethanol (%)	Time (min)	Temperature (°C)	Experimental Result	Predicted Value
73.5	38.3	62.5	101.7 ± 1.7	101.1

**Table 4:** Independent variables and their coded and actual values used for optimization.

Independent Variable	Symbol	Coded Levels		
		−1	0	1
Concentration of ethanol (%)	X_1_	30	60	75
Time (min)	X_2_	20	40	60
Temperature (°C)	X_3_	40	60	80
